# Using in-situ small-angle scattering to reveal the structure and dynamics of supramolecular polymers

**DOI:** 10.1038/s41467-025-65010-9

**Published:** 2025-10-21

**Authors:** Martin Hollamby, Hiroki Hanayama, Shiki Yagai

**Affiliations:** 1 School of Chemical and Physical Sciences, Keele University, Staffordshire, UK; 2 Department of Applied Chemistry and Biotechnology, Graduate School of Engineering, Chiba University, Chiba, Japan; 3 Institute for Advanced Academic Research (IAAR), Chiba, Japan

**Keywords:** Supramolecular polymers, Organic molecules in materials science, Supramolecular polymers

## Abstract

Small-angle scattering (SAS) is widely applied to nanoscale soft and hard material systems but has found limited use in the emerging field of supramolecular polymers (SPs). Key benefits to the field include in-situ measurement of SP assemblies in solution and the monitoring of triggered changes in real time. Here we summarise SAS basics and offer advice on the application of SAS to SP systems. To demonstrate applicability and show the capability of more advanced contrast-variation and time-resolved measurements, various successful SAS experiments on SP systems are highlighted. With a flexible sample environment allowing SAS measurement concurrent with other advanced techniques, plus ever-improving access to high quality data and analysis approaches, we conclude that SAS should be a more routine component in the toolbox of SP researchers.

## Abstract (web summary)

Small-angle scattering is widely applied to nanoscale material systems enabling in-situ measurements, statistically significant analysis and the study of stimuli dependent dynamic changes. Here, the authors show how small-angle scattering techniques can be applied to the characterization of supramolecular polymers and complement other routinely used techniques.

## Introduction

Small-angle scattering (SAS) exploits the interaction between an incident beam and objects in a sample that are differently contrasted to the background^1,2^. Here, we focus on the use of X-rays (SAXS)^3^ and neutrons (SANS)^4^ as incident radiation sources. These techniques have found widespread use in chemistry and materials science, from the study of self-assembly^5,6^ and gelation^7^ in solution to the characterisation of nanoparticles^8^, explosives^9^ and commercial steels^10^. SAS data provides information on length scales of order 1–100 nm, making it suitable for characterising supramolecular systems with mesoscale structural features, specifically supramolecular polymers (SPs)^11–13^. SPs are assemblies in which relatively small molecules aggregate in one dimension through non-covalent bonds. As in most cases, SPs form in solution, which makes them particularly well-suited for in-situ characterisation, which is the norm for SAS techniques. Results obtained by SAS are statistically significant, as even the thinnest X-ray beam simultaneously averages the scattering signal from over 10^10^ objects at an observable volume fraction. As shown below, a well-designed SAS experiment can identify or verify the overall dimensions and shape of SPs, their number density, and interrogate their internal structure, local environment and interactions. Structural evolution as a function of time can also be observed.

Both SAXS and SANS have found use in the characterisation of SPs. However, considering the large literature body on SPs, papers that use SAXS or SANS for characterisation are rare. Many recent reviews on SP characterisation^14–17^ either omit or only briefly mention the use of SAS, citing few examples. The reason might be the perceived lack of access to SAS experiments or the difficulty to unambiguously interpret SAS data for the analysis of complex systems. We, however, think that SAS is an important tool to study SP systems. After introducing some SAS basics, we highlight several successful applications of SAS in SP systems, demonstrating the depth of information that can be obtained and how it can be used to further develop SP systems.

## SAS basics

Where a SAS measurement takes place depends on the radiation source. SAXS experiments may be carried out using lab-based equipment, but there are also many dedicated beamlines at synchrotron facilities. On the other hand, SANS requires a neutron source such as either neutron research reactors (e.g. ILL, France) or spallation sources (e.g. ISIS, UK). Interactive global maps of synchrotron facilities and of neutron beam instruments are available from lightsource.org^18^ and the IAEA Knowledge Portal^19^, respectively. Access to a SAXS or SANS beamline typically requires an accepted peer-reviewed proposal, but any researcher who has an interesting scientific question may apply, making SAS techniques uniquely accessible to all. Moreover, depending on the facility, travel and accommodation for local users, plus reasonable consumables requests, may be covered as part of the awarded beamtime, and ‘buddy systems’ such as the NEPHEWS twinning programme at ISIS, UK, aiming to support those with limited experience, are becoming more commonplace.

**Fig. 1:**
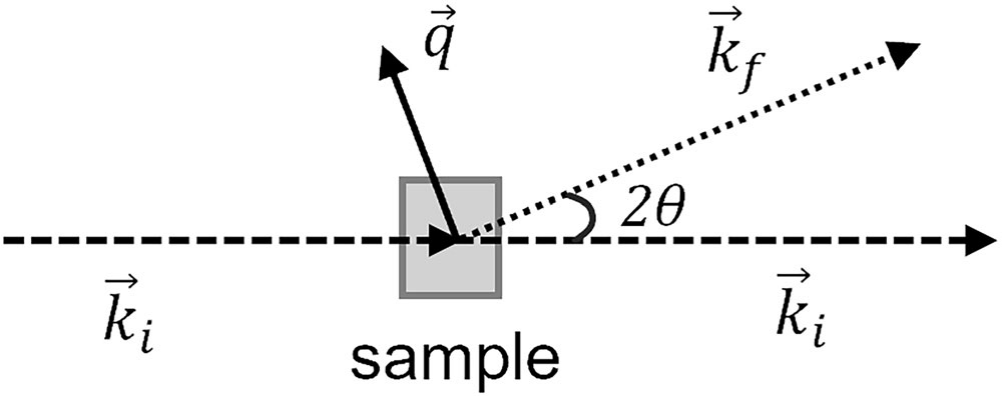
Small-angle scattering set-up. Typical SAS setup, showing the incident and scattered wave vectors, ${\vec{k}}_{{{\rm{i}}}}$ and ${\vec{k}}_{{{\rm{f}}}}$, and the scattering vector $\vec{q}$.

A typical SAS experimental setup is shown in Fig. 1. The incident beam $({\vec{k}}_{{{\rm{i}}}})$ of X-rays or neutrons passes through the sample and may be scattered $({\vec{k}}_{{{\rm{f}}}})$ by objects within it. Assuming elastic scattering, the magnitude of the scattering vector, is given as $Q=\left|\vec{q}\right|=\left|{\vec{k}}_{{{\rm{f}}}}-{\vec{k}}_{{{\rm{i}}}}\right|=4\pi \sin \theta /\lambda$ where *λ* is the wavelength of the incident radiation. In some studies, ‘*q*’ is used in place of ‘*Q*’, but has the same meaning.

SAS data is then typically plotted as scattered intensity, *I*(*Q*) versus *Q* (see later figures). *I(Q)* is generally described by Eq. 1, in which *P*(*Q*) is the form factor, which depends on SP size, shape and dispersity; *S*(*Q*) the structure factor, governed by local interactions or regular spacing within or between nearby SPs; *ϕ* is the SP volume fraction; *V*_SP_ the SP volume, and Δ*ρ* the difference in scattering length density between regions in the SP and the solvent.

1 $$I\left(Q\right)=\phi {V}_{{\mathrm{SP}}}\Delta {\rho }^{2}P\left(Q\right)S\left(Q\right)$$

Some discussion of available *P*(*Q*) and *S*(*Q*) models, and of other ways of analysing SAS data, is given below in the data analysis section. For a more detailed discussion of SAS theory, the reader is pointed to the many excellent books and review articles already published (e.g. refs. ^1–4,7,20–22^). In particular, ref. ^7^ on the related topic of supramolecular gels is recommended for beginners to the technique as it provides an excellent overview of many SAS basics.

The upshot of Eq. 1 is that for a given SP assembly observed at a given *ϕ*, with dimensions lying within the ~1–100 nm length scale probed by SAS, *I(Q)* is determined by Δ*ρ*. This value, explained in more detail in the next section, is dependent on the incident radiation used and on the composition of the SP.

## SAS contrast and contrast variation

A key difference between SAXS and SANS lies in Δ*ρ*. For X-rays, *ρ* is related to the electron density. For example, elements with a higher atomic number, and more electron-rich regions of organic molecules (e.g. aromatic or oxygen-rich regions) would have relatively high contrast in comparison to a less electron-dense solvent^1^. For neutrons, the magnitude of *ρ* is less clearly tied to atomic number, but large Δ*ρ* can be observed between element isotopes and particularly between regions rich in ^1^H and ^2^D^1,4^. Consequently, the SANS signal from an SP dispersed in a perdeuterated solvent is considerably stronger than that obtained from the same SP dispersed in the equivalent hydrogenated solvent. The SAXS signal arising from these two systems is expected to be the same, as deuteration does not alter electron density. Running SAXS on both samples allows any effect of deuteration on SP assembly to be checked, which may be important if comparisons between results obtained with deuterated (typically SANS, NMR) and non-deuterated solvents (most other techniques) are being made.

Individual scattering length densities, *ρ* of part of the SP system (e.g. solvent, part or whole of SP monomer) can be approximated using online calculators^23^ or those built into SAS analysis software packages. Inputs are mass density and empirical formula. Mass density is sometimes known or measured, but may otherwise be approximated, for example, using ACD/Chem Sketch freeware.

One implication of the above is that the SAXS and SANS data arising from an SP system in a perdeuterated solvent whose internal structure comprises regions with different compositions should differ. A non-aqueous example of this is an SP comprising a central electron-dense (e.g. aromatic, oxygen-rich) region surrounded by a shell of alkyl chains dispersed in a perdeuterated alkane solvent. This represents one of the most actively studied classes of SPs in current research. The Δ*ρ*_SAXS_ for the solvent vs. the alkyl chains is very low, so the alkyl region is almost invisible to SAXS, while Δ*ρ*_SAXS_ for the solvent vs. the electron dense region is high, and so the SAXS signal describes the core structure. Conversely, the Δ*ρ*_SANS_ for the solvent vs. the alkyl chains is very high, so the SANS signal may either describe only the shell or the whole SP assembly, depending on the core *ρ*_SANS_^24–26^. The SANS signal may be further attenuated by the use of mixtures of perdeuterated and hydrogenated solvent and/or by selective deuteration of parts of the SP structure to match *ρ* with that of the solvent. These advanced methods are referred to as ‘contrast variation’ or ‘contrast matching’ methods and are a unique feature of the SANS technique when applied to organic systems. Combined analysis of SAXS and (CV-)SANS data can allow for near-complete in-situ characterisation of SP solution structures.

**Fig. 2:**
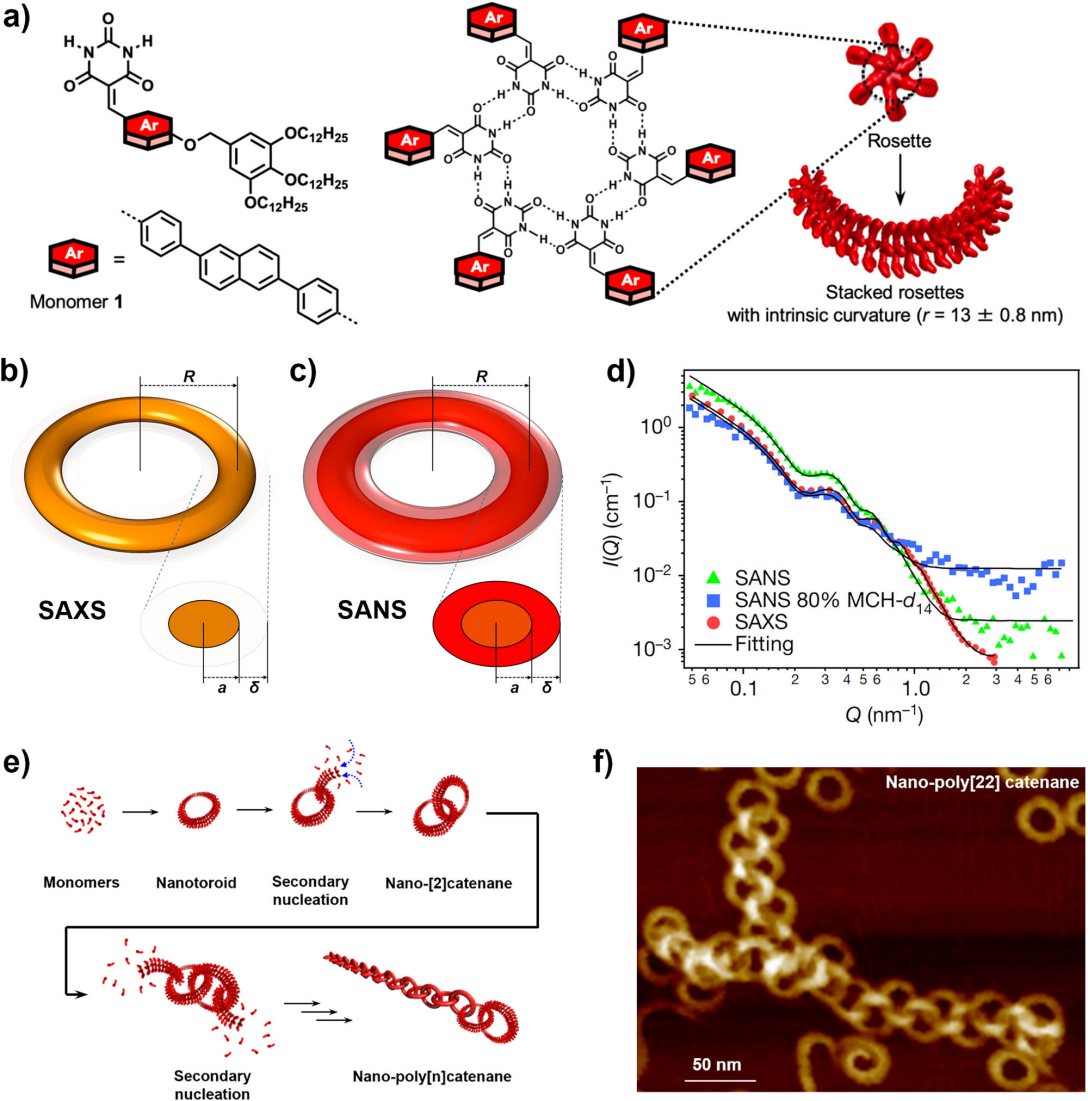
Contrast variation SANS and SAXS investigation of toroidal SPs and nano-poly[*n*]catenanes formed by barbiturated naphthalene derivatives. **a** Structure of **1** and schematic representation of how it forms hydrogen-bonded hexamers, which then stack to form the toroidal assemblies. **b** Schematic representation of toroidal assembly as visualised by SAXS—almost the entire scattering signal derives from the aromatic core (*ρ*_SAXS,core_ ~12 × 10^6^ Å^−2^; *ρ*_SAXS,shell_ ~7 × 10^6^ Å^−2^ *ρ*_SAXS,solvent_ ~7 × 10^6^ Å^−2^). **c** Schematic representation of toroidal assembly as visualised by SANS—the whole structure is observed (without solvent penetration, *ρ*_SANS,core_ ~2 × 10^6^ Å^−2^; *ρ*_SANS, shell_ ~−0.4 × 10^6^ Å^−2^ *ρ*_SAXS,solvent_ ~6 × 10^6^ Å^−2^). By allowing *ρ*_SANS, shell_ to float during analysis, the degree of solvent penetration was ascertained. **d** Fitted SAS data for the three contrasts. Data were analysed simultaneously, with structural parameters *R*, *a* and *δ* constrained to the same values. **e** Scheme showing the secondary nucleation process by which nano-poly[*n*]catenanes are formed and **f** AFM image of nano-poly[22]catenane^24^.

An example of this from our own work on SP systems is shown in Fig. 2. SAXS and CV-SANS measurements of the same solutions of toroidal SP structures were obtained^24,25^. It is notable that *I(Q)*_SANS_ decays to the background level at a lower *Q* than *I(Q)*_SAXS_, suggesting that the cross-sectional radius observed by SAXS (*a*) is smaller than SANS (*a* + *δ*). The simultaneous analysis of both datasets found globally consistent values of electron-rich aromatic core radius, *a*, and alkyl shell width, *δ*. Analysing *ρ*_shell_ with fixed *ρ*_core_ and *ρ*_solvent_ evidenced significant solvent penetration into the alkyl shell: the Δ*ρ* for the shell vs. solvent was smaller than expected, which can only be explained by the mixing of the ^2^D-rich solvent and ^1^H-rich alkyl chains. This suggested the alkyl shell as a potential location for the secondary nucleation required to form extensive nano-poly[*n*]catenanes and was corroborated by computational studies. With this insight, and by careful manipulation of the preparation method using sequential addition of monomers, nano-poly[*n*]catenanes comprising up to 22 rings were attained^24^.

## Data analysis methods and software

Data analysis is an important consideration in SAS experiments, and several suitable methods exist^1,4,7^. Many large-scale facilities hold data analysis workshops aimed at beginner users and some have dedicated data analysis support staff. Online resources are available, including the recently developed ‘SAStutorials.org’ website^27^. Software packages for SAS data analysis, introduced below, are now widely available and mostly free of charge.

Model fitting is a common analysis method. Many software packages are available, including standalone programmes such as SASfit^28^ and SasView^29^, while Igor Pro plugins are also available, including the NCNR SANS package^30^ and Irena^31^. An extensive range of form factor, *P*(*Q*) models may be deployed, ranging from basic geometric shapes (e.g. spheres, cylinders) to far more complex system specific models such as those representing multi-walled vesicles, star-shaped polymers or toroids. Parameters within these models may be monodisperse or distributed following several known distribution forms. Structure factors, *S*(*Q*) may be included where charged, aggregated or crystalline scattering objects are present. Key to SAS analysis by model fitting is model choice, as SAS cannot unambiguously determine both shape and size distribution from a single dataset^4^. Some analysis parameters must also be constrained during analysis to avoid erroneous unphysical conclusions. Here, prior knowledge of anticipated assembly shape and structure from complementary techniques is useful. Additionally, some parameters may be estimated. For example, Tanford’s formula (${l}_{{{\rm{c}}}}=1.5+1.265$ ${n}_{{{\rm{c}}}}$ where ${n}_{{{\rm{c}}}}$ is the number of carbons in the alkyl chain) can be used to predict alkyl chain lengths^24,32^.

**Fig. 3:**
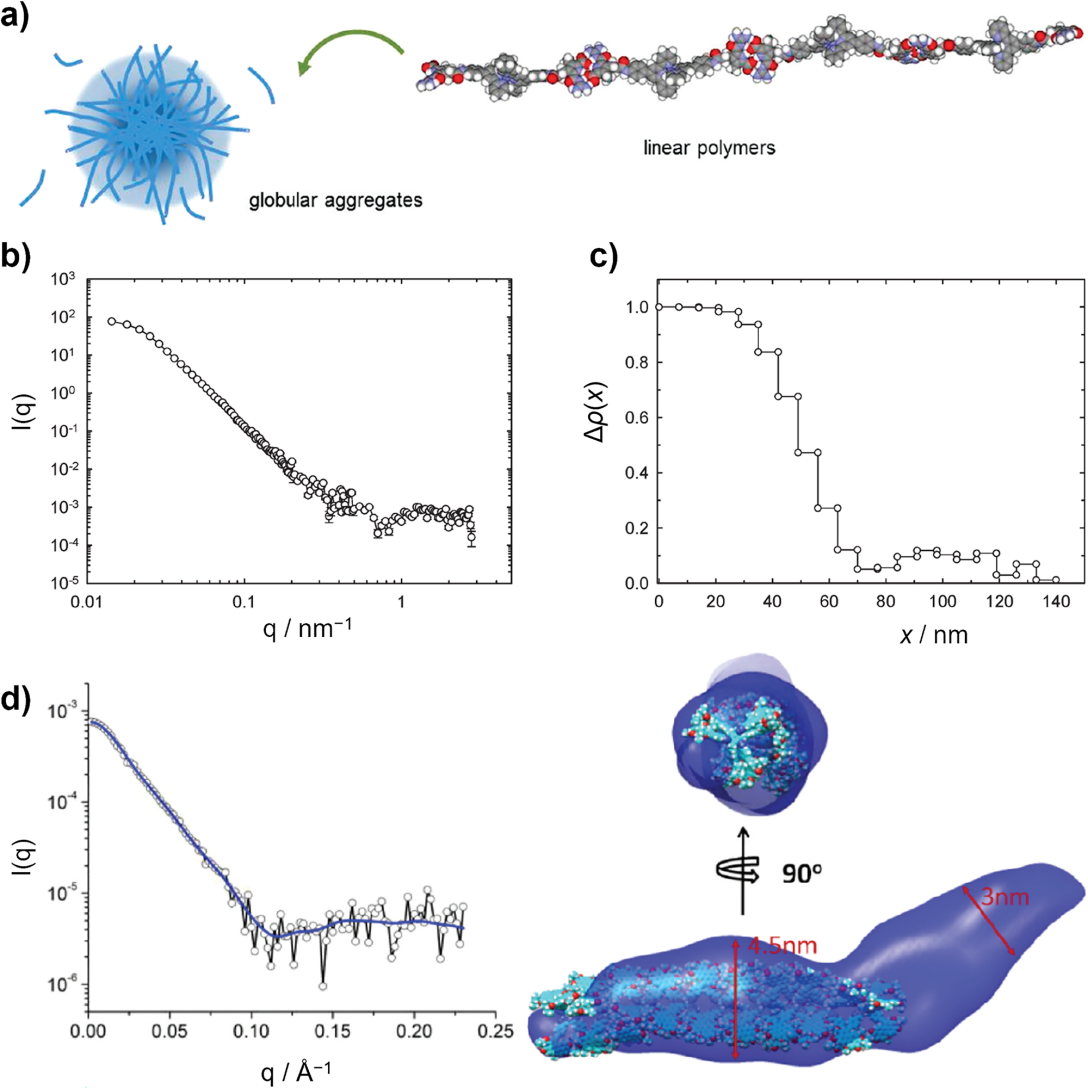
SP aggregates formed from linear polymers composed of metal-ligand and ion-pair bonding monomers. **a** Schematic representation of the formation of globular aggregates from the linear polymers described in ref. ^35^. **b** SANS data arising from the globular aggregates^35^. **c** Corresponding radial density profile Δ*ρ*(*x*), showing the higher density core and lower density shell of the aggregates^35^. Reprinted with permission from ref. ^35^. Copyright 2011 American Chemical Society **d** SAXS data obtained at 1 × 10^–5^ M in water/THF (70:30, v/v) arising from SPs formed from a hexa-substituted benzene scaffold decorated with perylene diimides as outlined in ref. ^37^. The DAMMIN fit is shown as a blue solid line. The molecular envelope output from the DAMMIN approach is shown on the RHS overlapping a molecular model obtained by molecular mechanics optimisation^37^. Reprinted with permission from ref. ^37^. Copyright 2011 American Chemical Society.

Other SAS data analysis methods are available, including the Indirect Fourier Transform (IFT) by Glatter^33^, which outputs a pair distance distribution function, or distribution form-free Monte Carlo methods such as those used by the standalone McSAS software^34^. These can be powerful, ‘assumption-free’ ways to determine information from SAS data. For example, SANS was used to elucidate the internal structure of aggregates formed by heteroditopic metal-ligand and ion-pair bonding monomers (Fig. 3a)^35^. SANS data is shown in Fig. 3b. Analysis using the IFT method backed up the presence of the aggregates >200 nm. The radial density profile (Fig. 3c) plots Δ*ρ* from the centre of the aggregate core (*x* = 0 nm). This shows two clear regions, with a dense core of ~30 nm (Δ*ρ* = 1) surrounded by a more open and solvent-swelled shell with a width of around 100 nm (e.g. as depicted in Fig. 3a)^35^.

Arguably the most advanced approach to SAS analysis combines SAS data with computational modelling of an assembly structure. This includes the DAMMIN approach^36^, which reconstructs a structural envelope from SAS data using a ‘dummy atom’ model, shown in Fig. 3d^37^. Other powerful methods use Monte Carlo or Molecular Dynamics simulations of a SP structure to simulate SAS data^38^. There, the minimisation process may be reversed to improve the agreement between simulation and data, provided that the system is sufficiently monodisperse. DAMMIN is part of the ATSAS software package^39^, which includes programmes for processing, visualising, analysing and modelling SAS data mainly arising from biological macromolecules, but as shown in Fig. 3 can also be used for data arising from SP systems. Recent advancements in the molecular modelling of SPs^40^ will only increase the applicability of these approaches.

**Fig. 4:**
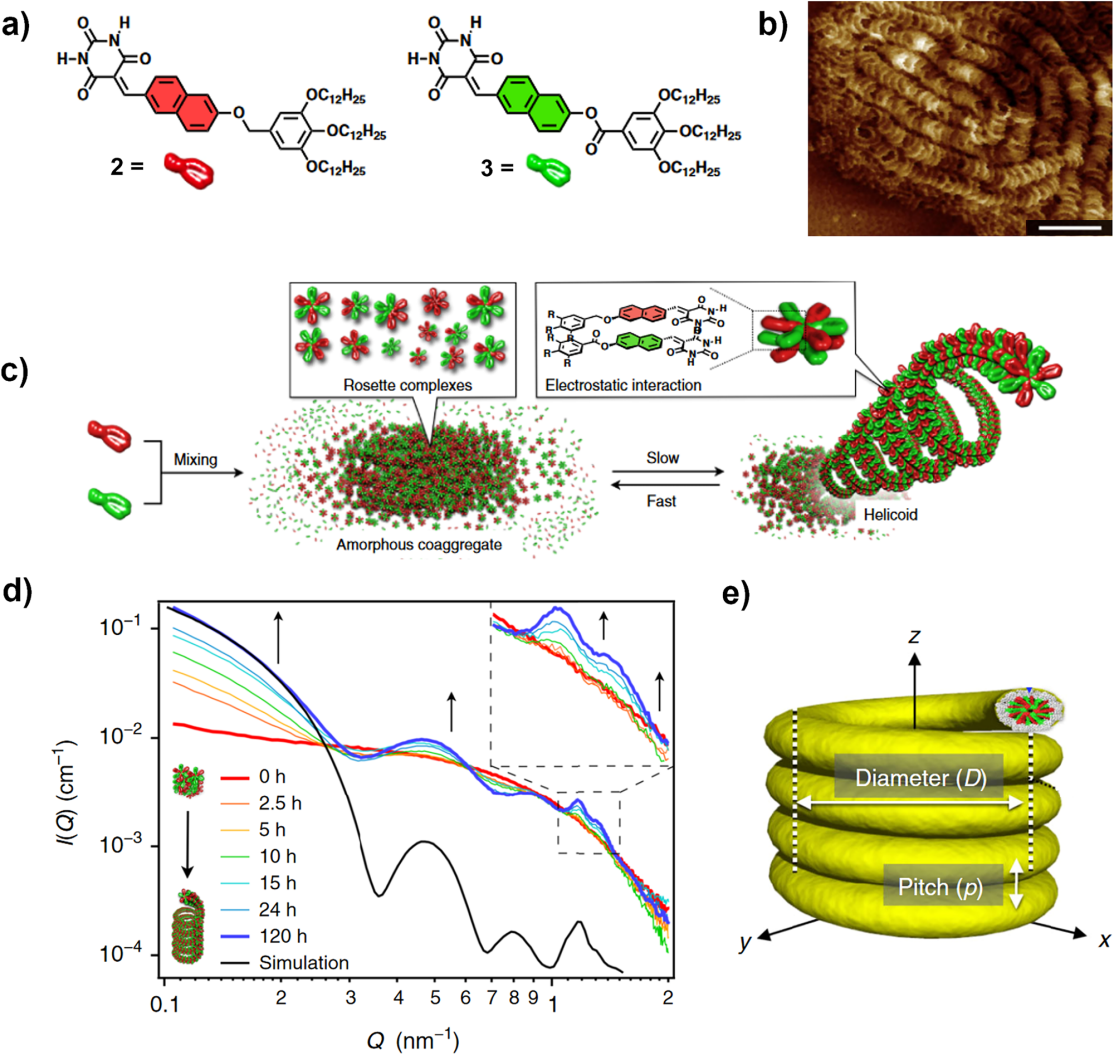
Helicoidal SPs formed by barbiturated naphthalene derivatives. **a** Chemical structures of monomers **2** and **3**. **b** AFM image of the helicoids (aged 31 h) formed by a 1:1 mixture of **2** and **3** (*c* = 100 μM) at 20 °C. Scale bar is 100 nm. **c** Schematic depiction of the helicoid assembly process. **d** Time-resolved SAXS data showing the growth of the helicoids from the amorphous coaggregates alongside the SPONGE simulation result. **e** Structure of helicoid, showing key parameters *D* and *p* investigated using the SPONGE^42^.

Related to the above is the SPONGE^41^. This simulates a SAS pattern from an 3D model input as an STL file drawn using any 3D-model making software otherwise often used for 3D-printing (e.g. CAD). As such, the software can in principle simulate a pattern from an assembly of any shape. In our own work, shown in Fig. 4, SAXS was used to verify the existence of helicoidal SP assemblies (Fig. 4c) in solution, formed from mixed naphthalene monomers **2** and **3**, with structures shown in Fig. 4a and imaged by ex situ AFM (Fig. 4b)^42^. The in-situ time-resolved SAXS data (Fig. 4d) monitored helicoid growth and developed a number of very well-defined features. By systematically varying individual structural parameters (e.g. Fig. 4e) using the SPONGE we were able to observe the effect on the resulting SAXS pattern of altering the pitch (*p*), the centre-to-centre diameter (*D*), the persistence length and even dynamic coil motion (i.e. the pitch changing in a sinusoidal manner)^42^. While the SPONGE simulation is at present limited to a single, sharp contrast step, which explains the imperfect intensity fit between simulation and data observed in Fig. 4d, it is a particularly flexible approach. With further method development, it is possible to see a more accessible future in which analysis of SAXS arising from complex structures might be limited only by the capability of the researcher to draw an accurate STL file.

## Complementary characterisation techniques

Regardless of the method used for SAS data analysis, the inclusion of information from other techniques is always advisable. In almost all cited examples in this article, SAS analysis is informed by complementary data. While spectroscopy^43^ and rheometry^44^ can provide useful information, and other scattering techniques such as wide-angle X-ray scattering, WAXS and X-ray diffraction, XRD, are invaluable for systems containing crystalline regions^45^, imaging techniques such as atomic force or electron microscopy (AFM, EM) are particularly complementary. The visual confirmation of SP structure that microscopy affords may inform and constrain SAS analysis, while the disadvantages of microscopy noted below (e.g. static, often ex-situ measurement, statistically small sample size, contrast difficulties) may be counteracted by SAS. For example, in one study cryo-EM imaging found a hexa-substituted benzene scaffold decorated with amphiphilic perylene diimides to form fibre-like assemblies, while SAXS analysis evidenced their presence in unfrozen solution^38^. In our own work, we use a wide range of complementary information to inform SAS analysis, including comparisons with AFM images of SP assemblies adsorbed onto HOPG substrates, and comparisons with computational methods^24,25,42,46–53^.

Given the numerous options above for SP characterisation, why bother to use SAS? Microscopy (AFM, EM) is effective for detailed observation of a small number of SP structures, but caution is required in assuming they reflect the overall state of SPs in a system. Recent studies have highlighted metastable assembly states and supramolecular polymorphism^51,54–58^, suggesting that conclusions drawn from limited observations on substrates can be risky. Microscopy also lacks the immediate capacity to capture a distribution of assemblies across an entire system. Statistically significant averaged results can be attained through analysis of regions in multiple samples, but that can be laborious. Elsewhere, absorption, fluorescence, and CD spectroscopic measurements may offer information on the average association states of molecules in the system containing π-electron-rich chromophore units. However, this typically reflects localised nanoscale structures involving a few molecules and may not capture mesoscale ordering. While accessing SAS equipment may itself take time, SAS complements many of the above limitations by providing in-situ information on larger-scale structural organisation. Deviations in scattering profile model fitting may indicate underlying structural insights, potentially due to the presence of supramolecular polymorphism. More significantly, dynamic changes in response to a wide range of internal or external triggers may be directly monitored using SAS, as noted in later sections.

## Applications of SAXS in SP systems

Of the two SAS techniques, SAXS is more commonly applied to SP systems, which may in part be due to ease of access to SAXS equipment. The inability to distinguish low electron density regions of SP monomer units from the solvent background can limit data analysis, but many groups, including our own, have attained useful information from SAXS data^36,38,42,46–53,59–66^.

**Fig. 5:**
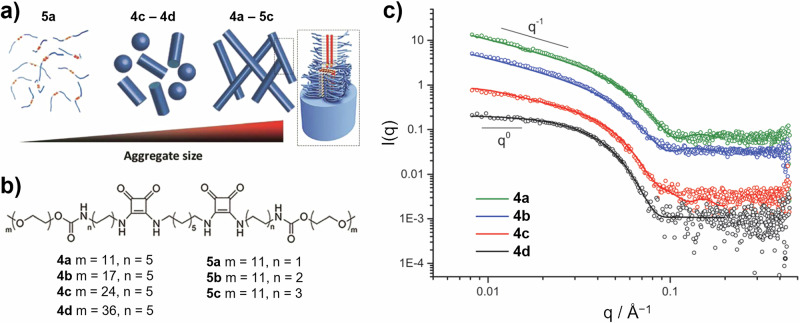
SAXS characterisation of structural change in squaramide-based SP assemblies. **a** Schematic representation of the change in structure of SP assemblies formed by the different monomers **4a**–**4d** and **5a**–**5c** shown in (**b**). **c** SAXS data arising from 4 mg mL^−1^ solutions of monomer units **4a**–**4d**^67^. The solvent-subtracted scattering profiles have been shifted vertically by multiplying by a factor of 2 (red line), 7 (blue line) and 20 (green line). Reproduced from ref. ^67^ with permission from the Royal Society of Chemistry.

In one example, SAXS was used to look at the effect of changing the lengths of hydrophobic central blocks and outer hydrophilic chains of squaramide-based monomers on the resulting SP assemblies (Fig. 5)^67^. Here, the SAXS contrast arises primarily from the squaramide core of the monomers (Fig. 5b). A large amount of information can be obtained by visual inspection of the SAXS data (Fig. 5c) prior to any quantitative analysis. The main observation is the change in the *I(Q)* dependency at low *Q*. According to SAS theory, rod-like species should exhibit a region in mid-low *Q* where *I(Q)* ~ *Q*^−1^, while non-interacting spherical species typically exhibit a region in which *I(Q)* appears to be independent of *Q* (i.e. *I(Q)* ~ *Q*^0^)^4,7^. Such behaviour is clearly observed in Fig. 5c, given strong evidence for a shift from spherical to rod-like assemblies as the length of the hydrophilic block decreases. Analysis using model fitting informed by cryo-TEM observations supported this (lines on Fig. 5c). The solution of **4d** comprises spherical assemblies with average radii of ~5.5 nm, while **4a** comprises long rods with radii of ~3.5 nm. Rod-like assemblies of **4a** and **4b** were found to comprise around 20 monomer units per nm along the length of the rod^67^.

As an aside, regions in SAS data where *I(Q)* ~ *Q*^−1^ or *I(Q)* ~ *Q*^0^ may be used to obtain size information using Guinier analysis. For example, for non-interacting spherical scatterers, this involves a plot of ln[*I(Q)*] vs. *Q*^2^, for which the straight-line gradient, *m* in the region *Q* ∙ *R*_*g*_ ≪ 1, is related to the radius of gyration, *R*_*g*_ of the scattering sphere as *m* = −*R*_*g*_^2^/3^1,3,4,20^. Other related methods include plotting log[*I(Q)*] vs. log[*Q*] to extract the fractal dimension of the scattering objects, and Zimm or Kratky plots, used to obtain information on the aggregation behaviour or chain confirmation of polymers in solution^7^.

**Fig. 6:**
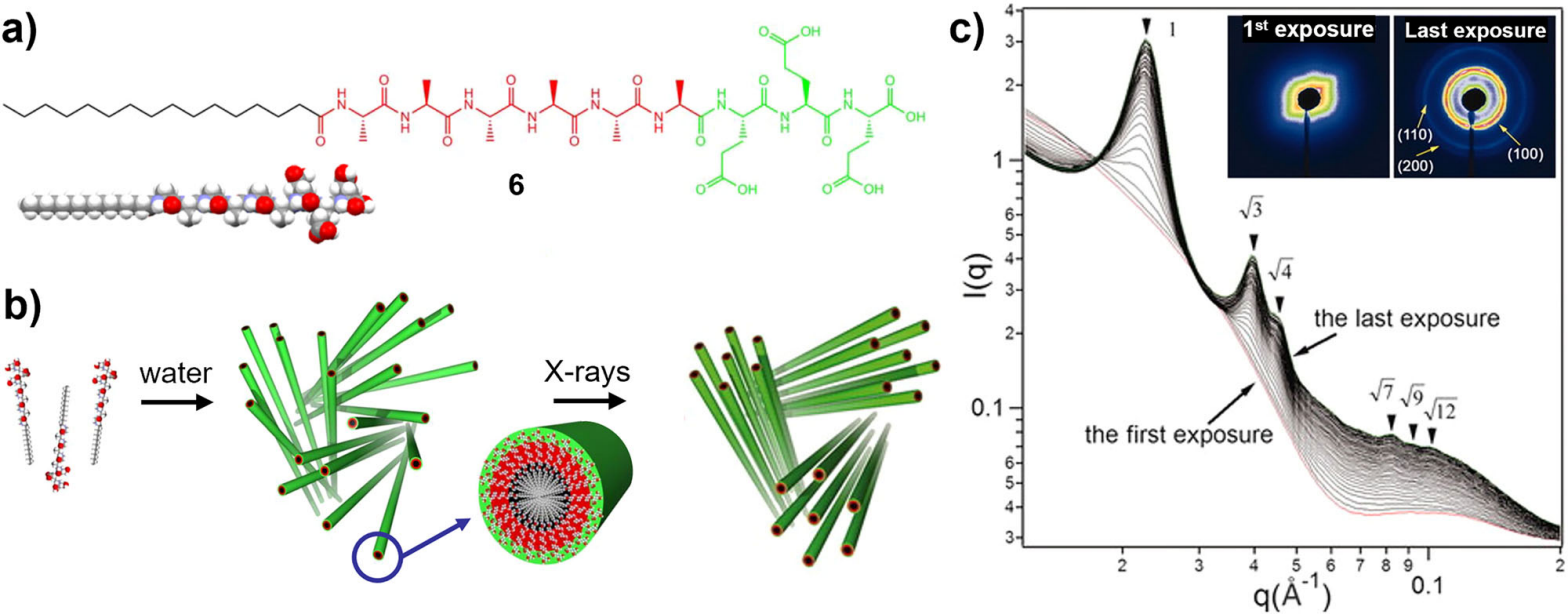
X-ray induced SP filament assembly. **a** Chemical structure and calotte model of oligopeptide **6**. **b** Schematic depiction of the assembly of **6** into filaments in water, and their X-ray induced ordering into a hexagonally packed system. **c** Time-resolved SAXS measurements showing the increase in hexagonal order (as shown by the increase in intensity and improved clarity of the labelled peaks) with increased exposure to the X-ray beam. From ref. ^70^. Reprinted with permission from AAAS.

One downside of the use of X-rays to probe SP systems, particularly using high flux synchrotron sources, is the potential for beam damage caused by the interaction between the ionising radiation and the electrons within the scattering objects. This can be counteracted using flow-through capillaries, a motorised sample environment and summing the signal from multiple short measurements from different sample positions (e.g. moving backwards and forwards along a capillary)^68,69^. However, under certain conditions, the powerful X-ray beam may trigger unusual and informative structural transformations. When SAXS was performed on SP filaments formed by oligopeptide **6** in dilute aqueous solution (0.5 wt%), diffraction patterns corresponding to the hexagonal packing of the filaments emerged and intensified with the number of measurements (Fig. 6), indicating a disorder-to-order transition (crystallisation)^70^. When the X-ray irradiation was stopped, the filaments returned to a disordered state within about 40 min. At higher solute concentration (e.g. 1+ wt%), hexagonal packing was observed even without X-ray irradiation, so this X-ray-triggered response occurs only in dilute solutions. Thermal effects were ruled out as the cause of the observed structural transition, as X-ray-triggered crystallisation was not observed at 40 °C. It is instead thought that X-ray irradiation induces the deprotonation of a small percentage of COOH groups on the surfaces of individual filaments, increasing electrostatic repulsion and promoting crystallisation. This is a very rare example, but the potential for such unique behaviour should be considered when designing and analysing results from a SAXS experiment.

## Applications of SANS in SP systems

**Fig. 7:**
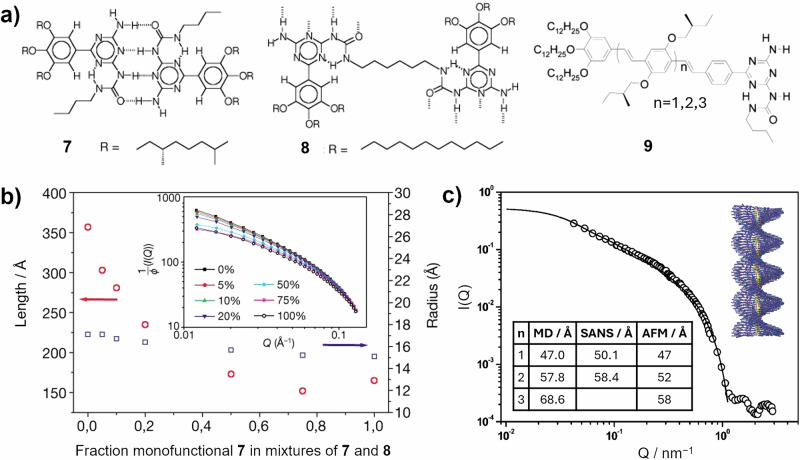
SANS characterisation of cylindrical and helical SPs. **a** Chemical structures of derivatives **7, 8** and **9**^77,78^. **b** (main panel) A plot of the length of the assembled structures estimated by SANS as a function of the fraction of **7**. (Inset) SANS arising from mixtures of **7** and **8** as a function of the fraction of **7** (expressed as a %). **c** SANS data arising from a solution of **9** with *n* = 2 in deuterated dodecane (overall *c* = 0.5 wt%)^77^. (Main panel) Change in length and radii values obtained by SANS analysis as a function of fraction of **7**^77^. Reproduced from ref. ^77^ with permission from Springer Nature (**c**) (inset table) Comparison of the diameter of the helical/cylindrical structures obtained by molecular dynamics simulation (MD), SANS and atomic force microscopy (AFM)^78^. **c** (inset, top right corner) SP structure formed by **9** with *n* = 2, determined by MD simulations^78^. Reprinted with permission from ref. ^78^. Copyright 2003 American Chemical Society.

SANS has also found application in SP systems^35,43,44,71–76^, although in comparison to SAXS its application is far less widespread. This may again be due to perceived difficulties of access, as the tuneable high contrast and lack of beam damage make SANS particularly suitable for studying SPs. In one example, shown in Fig. 7, SANS has been applied to SPs formed through the stacking of disk-shaped dimers of ureidotriazine derivative **7** (Fig. 7a) with quadruple hydrogen bonds, and bifunctional derivative **8** in deuterated dodecane (dodecane-*d*_26_)^77^. Data was collected on individual solutions of **7** and **8**, respectively, and modelled as arising from a cylindrical-like structure. Monofunctional **7** forms cylinders with cross-sectional radii of 15 Å and length of 100 Å, the latter of which increases with concentration. Bifunctional **8** forms cylinders with a slightly larger cross-sectional radii of 17 Å, reflecting the more extended longer R groups. SANS arising from mixtures of **7** and **8**, shown in Fig. 7b alongside the associated analysis results, shows a gradual change in radius from 17 to 15 Å with increasing fraction of **7**. However, a far greater change is noted in the column length, which falls abruptly on adding a relatively small fraction of **7**. This explained the Cotton effect noted by circular dichroism spectroscopy: **7** acts as an end capper, shortening the polymer chains formed in the mixtures^77^.

Elsewhere, SANS was applied to helical SPs of hydrogen-bonded dimers formed from extended π-conjugated monomers **9** (Fig. 7a)^78^. Here, SANS was used alongside molecular dynamics (MD) simulations and AFM microscopy to build a very detailed picture of the SP structures formed (e.g. inset Fig. 7c for **9** with *n* = 2). Example data for **9** with *n* = 2 is shown in Fig. 6c, the analysis of which, despite using a simplified model representing homogeneous cylindrical structures, gave parameters that showed very good agreement with results from the other techniques^78^.

A key benefit of SANS lies in the ability to perform contrast variation or matching experiments (e.g. CV-SANS), which can selectively highlight different parts of the whole SP structure. One example of this is shown in Fig. 2 and is explained above. That, however, uses only the change in ^1^H/^2^D ratio in the solvent mixture to affect the variation in contrast. In related other fields, for example, in studying the self-assembly of surfactants^6^ or low molecular weight gelators^7^, more advanced CV-SANS experiments involving the selective deuteration of assembling components are more routinely performed, allowing near-complete structural characterisation. The issue with applying such an approach to SP systems lies mainly in the difficult and costly synthesis of perdeuterated monomer units, although improvements in this area have recently been reported^79^. Advances here might stem from collaborative work between the SP community and dedicated deuteration facilities for neutron research that already exist globally, linked by the deuteration network (DeuNet)^80^.

## Time-resolved measurements

**Fig. 8:**
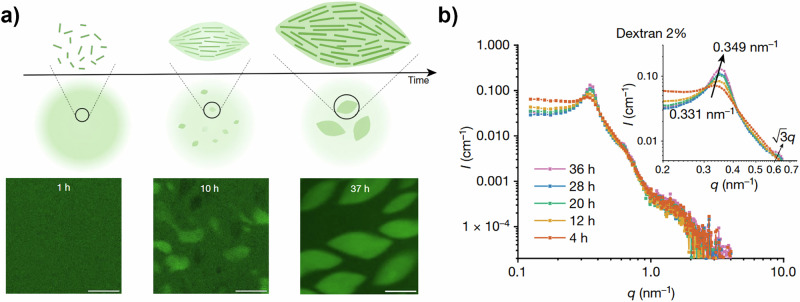
Emergence of internal structure within tactoid SP assemblies monitored by time-resolved SAXS. **a** Scheme showing the change in the ureidopyrimidinone glycine (UPy-Gly) supramolecular polymer system, from an initial homogenous dispersion of shorter fibrils (1 h) into liquid-liquid phase-separated tactoids upon fibril elongation (10 h) which grow and become denser with time (e.g. 37 h). Scale bars are 50 µm **b** SAXS profiles arising from the UPy-Gly system, including 2 wt% of dextran^83^.

The in-situ nature of SAS experiments opens up the possibility of monitoring the growth and dynamics of nanoscale systems as a function of time (TR-SAS). Time resolution may be on the order of milliseconds for SAXS and perhaps 100 s of milliseconds for SANS^81,82^, although in SP systems the lower concentrations often employed may limit these values. To-date, TR-SAS has found limited application in SP systems, although TR-SANS has been successfully used to monitor the evolution of structure in sonicated vs. non-sonicated gels, with a time resolution in the 10 s of minutes^75^. Elsewhere, other than the study presented in Fig. 7, TR-SAXS has tracked the emergence of the internal structure of liquid-liquid phase-separated tactoids formed within a dextran-containing ureidopyrimidinone glycine (UPy-Gly) SP system^83^. Fig. 8a shows the general scheme of spontaneous assembly, and Fig. 8b shows the TR-SAXS data for the system including 2% dextran. Here, the apparent main peak shift at *Q*_max_ ~ 0.34 nm^−1^ to lower *Q* and the appearance of a more resolved secondary peak both point to an increased uniformity within the tactoids over the time period (4–36 h) studied. The position of the secondary peak, at √3 · *Q*_max_, may point to a growing hexagonal ordering of the fibrils within the tactoids^83^.

## Summary and outlook

It is concluded that SAS techniques have much to contribute to the SP field. Looking to the future, we hope to see more complex in-situ and time-resolved experiments on SP systems, and more studies that take advantage of either the combined use of SAXS alongside SANS, or CV-SANS, as the additional contrast profiles considerably lower the ambiguity of data analysis.

In our own work, we are looking to further explore the potential of TR-SAS measurements, with the view to explore the dynamics of SP formation activated by mixing, changes in temperature or by the in-situ introduction of UV- and visible-light. Changes are being observed over a timescale of minutes. These experiments naturally use specialist equipment, including lightboxes, heating and cooling baths, and remote-controlled syringe pumps, but such sample environments are routinely applied at large-scale SAS facilities. Greater control over the precise activation time should allow faster processes to be visualised, as the scattering signal for each time frame may be averaged over multiple repeat measurements on the same system.

Using equipment already available at SAS facilities, other relevant opportunities arise. For example, the combined simultaneous measurement of SAXS + WAXS is routine in other fields and may allow simultaneous changes at the atomic and nanoscale to be probed, while simultaneous SANS + DLS may visualise assembly changes from 1 nm to over 1 µm. The combination of rheological measurements with SANS (e.g. Flow-SANS or Rheo-SANS)^84^, regularly used to study conventional polymer systems and supramolecular gels, may provide useful insight, particularly in more concentrated SP systems. Such a growth in use may require more routine SP polymer deuteration, but—as noted above—dedicated facilities already exist to support this type of synthesis. A final challenge is that improvements in analysis software model portfolios are required to accommodate, or to at least approximate, the complex shapes that SP systems might adopt. Here, the analysis of SAS data alongside simulation results might prove the solution, although other methods, such as the SPONGE may prove more accessible.
